# A bioinformatics pipeline for estimating mitochondrial DNA copy number and heteroplasmy levels from whole genome sequencing data

**DOI:** 10.1093/nargab/lqac034

**Published:** 2022-05-17

**Authors:** Stephanie L Battle, Daniela Puiu, TOPMed mtDNA Working Group, Joost Verlouw, Linda Broer, Eric Boerwinkle, Kent D Taylor, Jerome I Rotter, Stephan S Rich, Megan L Grove, Nathan Pankratz, Jessica L Fetterman, Chunyu Liu, Dan E Arking

**Affiliations:** McKusick-Nathans Institute, Department of Genetic Medicine, Johns Hopkins University School of Medicine, Baltimore, MD, USA; Department of Biomedical Engineering, Johns Hopkins University, Baltimore, MD, USA; Department of Internal Medicine, Erasmus Medical Center, Rotterdam, The Netherlands; Department of Internal Medicine, Erasmus Medical Center, Rotterdam, The Netherlands; Human Genetics Center, Department of Epidemiology, Human Genetics, and Environmental Sciences, School of Public Health, The University of Texas Health Science Center at Houston, Houston, TX, USA; Human Genome Sequencing Center, Baylor College of Medicine, Houston, TX, USA; The Institute for Translational Genomics and Population Sciences, Department of Pediatrics, The Lundquist Institute for Biomedical Innovation at Harbor-UCLA Medical Center, Torrance, CA, USA; The Institute for Translational Genomics and Population Sciences, Department of Pediatrics, The Lundquist Institute for Biomedical Innovation at Harbor-UCLA Medical Center, Torrance, CA, USA; Center for Public Health Genomics, University of Virginia, Charlottesville, VA, USA; Human Genetics Center, Department of Epidemiology, Human Genetics, and Environmental Sciences, School of Public Health, The University of Texas Health Science Center at Houston, Houston, TX, USA; Department of Laboratory Medicine and Pathology, University of Minnesota, Minneapolis, MN, USA; Evans Department of Medicine and the Whitaker Cardiovascular Institute, Boston University School of Medicine, Boston, MA, USA; Framingham Heart Study, Boston University School of Medicine, Boston, MA, USA; Department of Biostatistics, Boston University School of Public Health, Boston, MA, USA; McKusick-Nathans Institute, Department of Genetic Medicine, Johns Hopkins University School of Medicine, Baltimore, MD, USA

## Abstract

Mitochondrial diseases are a heterogeneous group of disorders that can be caused by mutations in the nuclear or mitochondrial genome. Mitochondrial DNA (mtDNA) variants may exist in a state of heteroplasmy, where a percentage of DNA molecules harbor a variant, or homoplasmy, where all DNA molecules have the same variant. The relative quantity of mtDNA in a cell, or copy number (mtDNA-CN), is associated with mitochondrial function, human disease, and mortality. To facilitate accurate identification of heteroplasmy and quantify mtDNA-CN, we built a bioinformatics pipeline that takes whole genome sequencing data and outputs mitochondrial variants, and mtDNA-CN. We incorporate variant annotations to facilitate determination of variant significance. Our pipeline yields uniform coverage by remapping to a circularized chrM and by recovering reads falsely mapped to nuclear-encoded mitochondrial sequences. Notably, we construct a consensus chrM sequence for each sample and recall heteroplasmy against the sample's unique mitochondrial genome. We observe an approximately 3-fold increased association with age for heteroplasmic variants in non-homopolymer regions and, are better able to capture genetic variation in the D-loop of chrM compared to existing software. Our bioinformatics pipeline more accurately captures features of mitochondrial genetics than existing pipelines that are important in understanding how mitochondrial dysfunction contributes to disease.

## INTRODUCTION

Approximately 1 in 8000 people are diagnosed with a mitochondrial disease caused by a mitochondrial DNA (mtDNA) mutation (1). Mitochondrial diseases are heterogeneous in their clinical manifestation and typically affect multiple organ systems (2). For example, Leigh syndrome, the most common childhood mitochondrial disease, can be caused by >75 different mutations in nuclear or mitochondrial genes (3). Some of the features include neurological symptoms, hypertrichosis, and dysmorphic features (2,3). MELAS, or Mitochondrial Encephalopathy, Lactic acidosis, and Stroke-like episodes, is a mitochondrial disorder where 80% of cases are caused by a mutation in a mitochondrial tRNA gene (4). Sequencing patient DNA is commonly included as part of the diagnosis of mitochondrial diseases; therefore, being able to assess multiple features of mitochondrial genetics from genome sequencing data will be of significant benefit to human health.

The mitochondrion is a ubiquitous organelle with complex genetics. Unlike the nuclear genome, which is only present in two copies, there can be ∼1000 to 10 000 copies of mtDNA in most somatic cells (5) and up to 150 000 in mature oocytes (6). The relative amount, or copy number, of mtDNA is associated with aging and overall-mortality (7,8). Additionally, the mitochondrial genome has a 17-fold higher mutation rate than the nuclear genome (9). Thus, the mtDNA can exist in a state of heteroplasmy, where there is variation in the sequence of the different mtDNA molecules within a cell, or homoplasmy, where all mtDNA share the same sequence. Pathogenic mutations in mtDNA are usually present in a heteroplasmic state, and the level of heteroplasmy is directly linked to mitochondria function (2). Both the quantity (as measured by copy number) and quality (as measured by heteroplasmic load) of mtDNA have been linked to disease (2,8).

There are several software packages designed to take whole genome sequencing (WGS) data and extract mtDNA for variant identification. MToolBox (10) can extract mitochondrial reads from WGS or whole exome sequencing (WES) data to identify heteroplasmic single nucleotide variants (SNVs), insertions/deletions (INDELs) and haplogroup information. mtDNA-Server (11) which uses the program Mutserve, identifies heteroplasmy and works very well on large datasets. MitoAnalyzer (12,13) performs both heteroplasmy calling and copy number calculations. Mity (14) is another software that detects heteroplasmy SNVs and INDELs from WGS data. These software attempt to address two basic features of mitochondrial genetics, sequence variation and copy number, and each has its own unique limitations. None of them attempts to recover sequencing reads at regions of low coverage, which is important for thorough variant discovery.

Here, we present a bioinformatic pipeline, named Mitochondrial High Performance Caller, referred to as MitoHPC, to estimate mitochondrial copy number (mtDNA-CN) and heteroplasmy from WGS samples. MitoHPC is able to obtain uniform coverage across chrM and remove contaminating nuclear-integrated mitochondrial sequences (NUMTs). MitoHPC also constructs a reference sequence for each sample and identifies heteroplasmic variants for each sample using its own reference. The pipeline additionally annotates SNVs and INDELs to allow for better identification of true variation from sequencing or mapping errors. MitoHPC is designed to be a useful tool for accurately quantifying mtDNA-CN and identifying heteroplasmy in tens of thousands of samples with short computational run times and minimal computational requirements. This makes MitoHPC ideal for large genomics datasets.

## MATERIALS AND METHODS

### Datasets

Datasets are from the Trans-Omics for Precision Medicine (TOPMed) program, freeze 8 (15). TOPMed studies provide WGS data at ∼30× genomic coverage using Illumina next-generation sequencing technology. TOPMed WGS data must pass specific quality control metrics before it is released for use by the scientific community. Additional information on TOPMed WGS data generation and processing can be found here: https://www.nhlbiwgs.org/data-sets

We analyzed WGS data from the Atherosclerosis Risk in Communities (ARIC) study (16) and the Multi-Ethnic Study of Atherosclerosis (MESA) study (17). Both ARIC and MESA are population-based longitudinal cohort studies with 3930 and 5370 WGS samples available, respectively. One sample in ARIC was excluded due to lack of proper consent. ARIC WGS samples were comprised of deep vein thrombosis and early-onset atrial fibrillation cases (<10% of dataset) and controls. In the ARIC study, DNA for WGS were isolated from buffy coat using the Gentra Puregene Blood Kit (Qiagen), The ARIC cohort is 52% female, age range 45–74 at time of DNA isolation with the following racial backgrounds: 93% European American and 7% African American. MESA participants were required to have no known clinical CVD upon recruitment. In MESA, DNA was isolated from peripheral leukocytes using the Gentra Puregene Blood Kit. The MESA cohort is 53% female, age range 45–84 with the following racial backgrounds: 38% European American, 28% African American, 22% Hispanic and 12% Chinese American ancestry.

For the MESA cohort, we were able to identify 559 poor quality samples. These samples had lower DNA quality due to a temporary change in the DNA extraction method used on samples extracted from November 2001 through December 2001. We removed these 559 samples from all analyses.

### TOPMed google cloud data access and extracting metadata

The TOPMed datasets used for our study were accessed using Google Computing Services (Supplementary Figure S1). Samples were processed in batches of 100. We downloaded CRAM and CRAI files using fusera, extracted chrM/NUMT reads using ‘samtools view –T hg38-reference-file chrM chr1:629084–634422 chr17:22521366–22521502’, and generated output BAM files.

### Processing the FASTQ files

**Figure 1.**
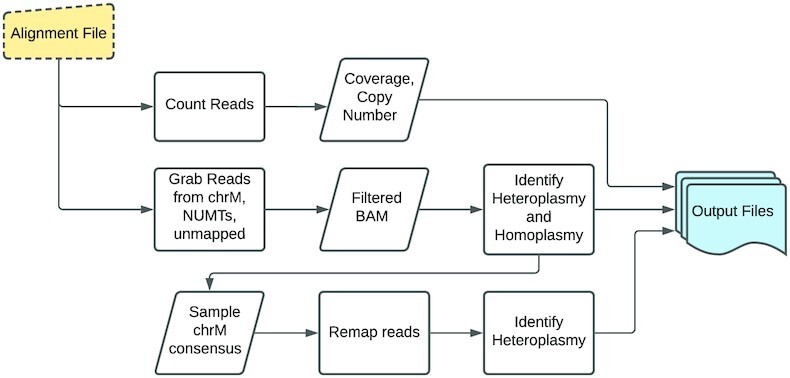
Overview of mtDNA-CN and Heteroplasmy Analysis Pipeline.

We designed MitoHPC to run on aligned human WGS data or mitochondrial enriched sequencing data provided as either BAM or CRAM file format (Figure 1). Prior to running MitoHPC, we trimmed and aligned FASTQ files to the whole human genome assembly using an aligner which generates SAM/BAM/CRAM output alignments. SAM format alignment files can be converted to BAM/CRAM format using SAMtools software (18). The alignment files were sorted (samtools sort) and indexed (samtools index). The aligned reads counts (samtools idxstats) were used for mtDNA-CN estimation (Supplementary Figure S1).

### Calculations for mtDNA-CN

The general calculation for mtDNA-CN is shown below, as used by Ding et al. (12) :

\begin{equation*}2 \times \left( {{\mathrm{chrM}}\,{\mathrm{coverage}}} \right)/\left( {{\mathrm{genome}}\,{\mathrm{coverage}}} \right)\end{equation*}

Mitochondrial genome coverage is calculated as the number of mapped reads to chrM times the read length divided by the length of chrM. Genome coverage is calculated using three different methods described below:

- coverage calculated by TOPMed from mappable reads passing data quality filters aligned to a sex specific mappable genome
- (mapped bases)/(3,031,865,587 bases in human genome)
- (mapped bases)/(3,054,815,472 bases if female) or (3,008,915,703 bases if male)

Genome sizes are taken from the Telomere-to-Telomere Consortium (19). The comparison of these different copy number metrics is described in the Results section.

### Extracting and re-mapping reads to a circularized chrM

**Figure 2.**
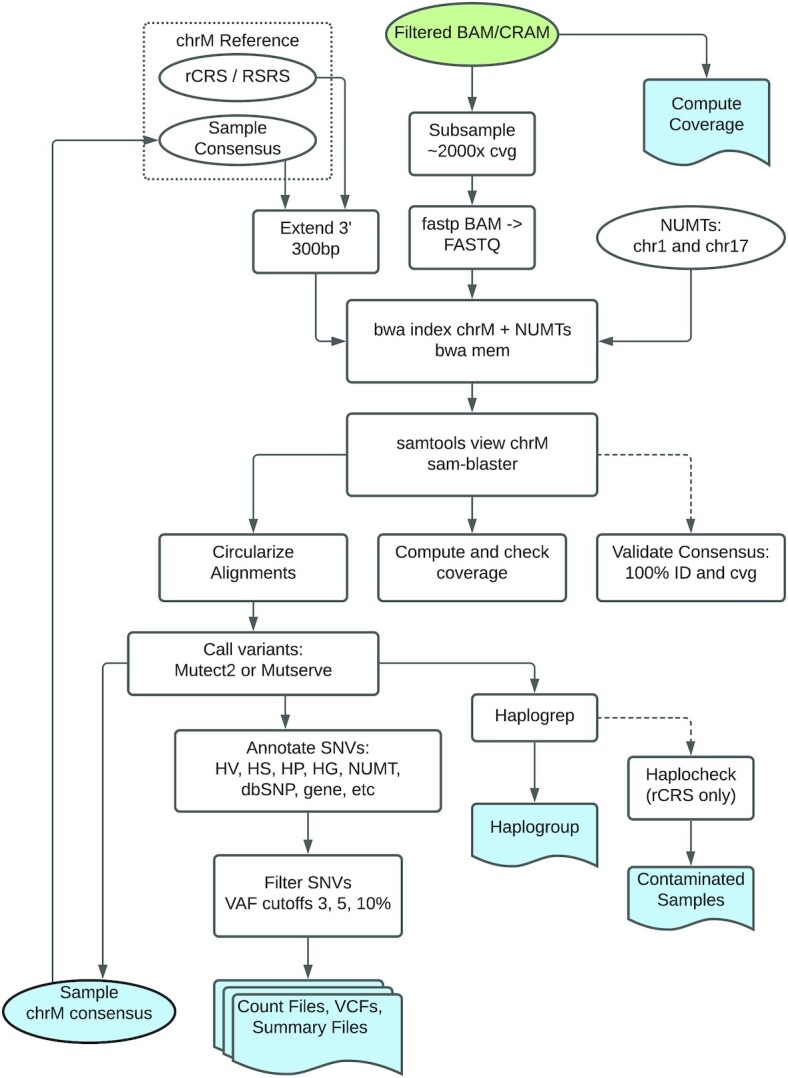
Detailed flowchart of pipeline processes. Input is BAM/CRAM file (green) at the top of the diagram. Outputs are shaded blue. The following abbreviations are used in the Annotate SNVs box and are described in detail in the Variant Annotation of the Methods section: HV, known hypervariable regions; HS, mitochondrial hotspots; HP, homopolymer region; HG, haplogroup specific; NUMT, the variant matches a known variant in the nuclear genome; dbSNP, the variant is found in the dbSNP database.

The WGS data was mapped to the human genome build GRCh38 (Figure 2). We used SAMtools to extract reads that mapped to the mitochondrial chromosome, chrM, and the NUMT regions (hg38 chr1:629084–634422 and chr17:22521366–22521502). These regions were identified as mitochondrial read ‘sinks’ by mapping mitochondrial reads to the nuclear chromosomes and identifying regions of read pile up. We also retrieved unmapped reads where one mate mapped to chrM. We remapped the reads to a circularized version of chrM with position mt16569 extended downstream 300 bases and to the NUMT regions on chr1 and chr17 (Figure 2). This extended end was included in the ref.fa file which was indexed using ‘bwa index’. The reads were trimmed using fastp (20) and remapped using BWA (21), ‘bwa mem *input.fa inputfile* -p -v 1 -t 1 -Y -R *headerline* -v 1’. Duplicate reads were removed using SAMBLASTER (22), ‘samblaster –removeDups –addMateTags’. Alignments that spanned the chrM start-stop were split and kept as two alignments.

### Detecting sample heteroplasmy, homoplasmy and haplogroup

Prior to running the variant calling software, we down-sampled the chrM reads for each sample to ∼2000× coverage (Figure 2). This increases the speed of MitoHPC while still retaining sufficient coverage to have confidence in low level (3% variant allele frequency) heteroplasmy calls. We incorporated two programs for calling mtDNA heteroplamic and homoplasmic variants, GATK Mutect2 (23,24) and Mutserve (11) (Figure 2). For the first iteration of variant identification, we used the rCRS as the reference genome, although the RSRS is included as an optional reference. Mutect2 was run using default parameters and the output VCF was run through FilterMutectCalls command with the following additional parameters: *–min-reads-per-strand 2*. Mutserve was run using the following additional parameters: *–deletions –insertions –level 0.01*. For both programs, we used a 3% variant allele frequency (VAF) threshold.

After the first iteration of variant identification, we run the program HaploGrep v2.4 (25) to identify mitochondrial haplogroups for each sample. Samples are assigned to a haplogroup based on the known variants in Phylotree17_FU1 tree (26).

### Variant annotation

Variant annotations are included in the VCF output files. In addition to annotations created by Mutect2, we included annotations that add genomic and biological context to the variant sites identified. The following annotation files are provided on GitHub (https://github.com/ArkingLab/MitoHPC/tree/main/RefSeq): (i) mitochondrial hypervariable (HV) regions; (ii) chrM homopolymer (HP) regions defined as five or more Cs in a row, one mismatch, ±1 bp on the ends; (iii) mitochondrial hotspot regions (HS) as described by Nussbaum (27); (iv) genic information like coding regions (CDS), D-loop and gene name; (v) manually curated database of 1098 haplogroup specific (HG) SNVs are those that occur in 80% of GenBank samples (*n* = 35 502) of the same haplogroup and not present in other haplogroups (Supplementary Table S1). SNVs were identified by aligning samples’ chrM reads to the rCRS, while haplogroup was determined using Haplogrep2; (vi) 382 NUMT SNVs identified by aligning the NUMTs we identified on chr1 and chr17 and published in Lutz-Bonengel *et al.* (28) and Dayama *et al.* (29) to rCRS using MUMMer (30) nucmer and show-snps (Supplementary Table S2); (vii) dbSNP variants in dbSNP database (31). We also annotate variants with APOGEE score (32) a measure of pathogenicity of mitochondrial variants.

### Generate consensus sequence and validate alignments

We use the program BCFtools (33) ‘bcftools consensus’ to generate a new mitochondrial consensus fasta sequence for each sample incorporating homoplasmies and major alleles from the Mutect2 output. This step generates the sample's unique mitochondrial reference sequence for the second iteration of heteroplasmy calling. This sequence was circularized (300 bp from position mt1 added after position mt16569) and indexed using BWA (21) ‘bwa index’. The 2000x coverage reads were aligned using ‘bwa mem’. Exact alignments (100% identity, 100% length) were converted to BED format and merged using BEDTools (34) ‘bedtools merge –d −5’, making sure the reference was fully covered.

### Contamination check

Due to the exclusive maternal inheritance, each sample should have only one dominant haplogroup detected. We ran Haplocheck (35) on samples after haplogroup identification. The Haplocheck output file lists all samples and contamination status. We removed samples with a contamination level of 3% or more from downstream analyses.

### Statistical analyses

Statistical analyses were performed using R version 4.0.4. To test for an association with age and mtDNA-CN, we ran a linear model adjusted for sex and collection center (site where sample blood draw took place) as a random effect. Self-reported race was not included as it did not significantly affect the model. The polygenic risk score (PRS) is included in the linear model for ARIC only. A binomial generalized linear model was used for dichotomized heteroplasmy data (where ‘0’ means no heteroplasmic sites and ‘1’ means at least one heteroplasmic site) and included the following covariates: age, sex, self-reported race, and collection center. Average heteroplasmy count was determined after removing outlier counts that were three or more standard deviations away from the mean.

## RESULTS

### Overview of pipeline

Our goal was to create a bioinformatics pipeline that incorporates multiple features of mitochondrial genetics to readily facilitate downstream analyses. MitoHPC includes four main parts: (i) calculation of mtDNA-CN; (ii) identification of heteroplasmic and homoplasmic variants against the rCRS (referred to as ‘first iteration’ of variant calling); (iii) generation of the sample specific chrM consensus sequence by incorporating homoplasmies and major heteroplasmic alleles using the rCRS as the backbone (referred to as ‘second iteration’ of variant calling) and (iv) re-calling heteroplasmy for each sample mapped using its own consensus sequence as the reference. MitoHPC produces three main outputs. The first output is a mtDNA-CN summary file with read count, coverage, and mtDNA-CN counts. The second output is a variant summary information file, which includes haplogroup, a count of mtDNA homoplasmic sites, heteroplasmic sites, SNVs, INDELS at all locations and at non-homopolymer regions. The third output consists of VCF files of the annotated heteroplasmic and homoplasmic sites. Re-calling heteroplasmy against a sample's own reference generates the additional summary and VCF files. The code is available on GitHub: https://github.com/ArkingLab/MitoHPC.

### Computational speed

We utilized Google Cloud to filter the chrM and NUMT reads from TOPMed samples. The sample alignment files were processed in batches of 30 on a single processor and took ∼2 min per sample to complete. Running in parallel with a maximum 240 jobs at one time, it took ∼1.5 days to process the 90K samples. When it comes to computational speed, our pipeline is designed to handle large genomics dataset of tens of thousands of samples quickly and cost-effectively.

### Recovering low coverage areas

**Figure 3.**
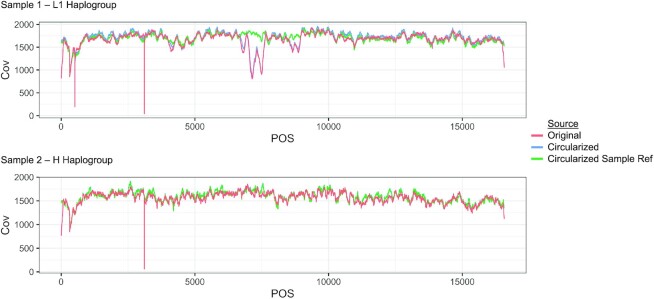
Coverage for two samples in ARIC plotted across the full length chrM. Sample 1 belongs to haplogroup L1 and Sample 2 belongs to haplogroup H. Reads were deduplicated prior to plotting. The red line is chrM coverage from the original alignment file. The blue line is the chrM coverage after aligning the reads to the circularized rCRS reference. The green line is the chrM coverage after realigning the reads to the sample's reconstructed chrM reference sequence. All reads were down-sampled evenly for plotting.

Accurately aligning reads to the chrM is a non-trivial task. First, chrM is circular, and commonly used aligners expect linear chromosomes. Second, chrM reads can falsely align to NUMTs in the nuclear genome. From the provided TOPMed metadata, we first checked the uniformity of coverage across chrM (Figure 3, red line). As expected, we saw noticeable dips at the ends of the chrM and at other sites known to have low coverage. Position 310 and 460 lie within polycytosine tracts (chrM:300–320 AAACCCCCCCTCCCCCGCTTC and chrM:450–470 TATTTTCCCCTCCCACTCCCA) and have previously been reported to have low coverage in sequencing data (36). Low coverage at three mitochondrial hypervariable regions due to homopolymer polycytosine tracts in these regions have also previously been reported (37). This is due to polymerase slippage at regions of low nucleotide complexity either during sequencing, library PCR, or within the cell during mitochondrial genome replication (36).

To recover reads at low coverage regions we started by realigning the reads to the circularized version of chrM. Due to the ‘edge’ effect (low coverage at the start/stop of chrM), and the similarity of the chr17 NUMT to chrM D-loop (bwa mem minimum alignment score of 30), the coverage in the D-loop region is about ∼40% that of the chrM median coverage (Figure 3, red line). In the first iteration of variant calling, MitoHPC aligns to the circularized rCRS and the average D-loop coverage increased to more than ∼90% of the average total chrM coverage (Figure 3, blue line). In the second iteration MitoHPC realigns reads to a sample's unique consensus chrM sequence, increasing the coverage of other low coverage regions that may be due to specific sequence variation in the sample. For example, in Figure 3, Sample 1 from ARIC had a major dip in coverage upstream of position 7500, which was only recovered when using the sample's unique consensus as a reference for alignment (Figure 3, green line). Sample 2 had a more subtle difference in coverage but still showed a notable increase in coverage at the start/stop of chrM. These 2 samples are from different haplogroups (L1 and H respectively) and they demonstrate how individuals from haplogroups that are more distant from the rCRS can have variation that causes uneven read coverage across chrM. Our pipeline prioritizes uniform chrM read coverage prior to heteroplasmy calling; however, it is still possible for some samples to have low average depth of coverage. It is important to inspect the coverage depth of outlier samples and variants. With our method of realigning to a sample's unique reference, we are able to attenuate large differences in coverage across chrM.

### Contamination check

We included the program Haplocheck (35) in MitoHPC to output the contamination status of each sample. Haplocheck identifies potentially contaminated samples by looking for the presence of common variants from more than one mitochondrial haplogroup. Four samples out of 3929 in ARIC and 10 samples of out 5370 in MESA had a Haplocheck contamination level of 3% or more. For our purposes, the potentially contaminated samples were excluded from our downstream analyses. Although depending on the biology of the samples in question, it may be worth investigating the ‘contaminated’ samples further. The inclusion of additional sample QC checks, like Haplocheck, in our pipeline allows for the user to easily identify samples of poor quality, as their results could confound downstream analyses.

### mtDNA-CN calculation comparisons

Mitochondrial DNA copy number is a metric commonly used for mitochondrial quantity in a cell or tissue and is associated with mitochondrial function (38). It is based on the ratio of mtDNA to nuclear DNA, calculated using the equation defined by Ding *et al.* (12) as two times the ratio of chrM coverage to genome coverage. The chrM coverage was relatively uniform across all haplogroups in ARIC and MESA cohorts (Supplementary Figure S2) except for main haplogroups L (includes groups L0-6; *P*-value 4.77 × 10^–7^) and R (includes groups R1-9, B, P F; *P*-value 4.08 × 10^–4^) in MESA. We calculated the genome coverage using three different approaches to determine whether the subtle differences in the mtDNA-CN calculation would have an impact (see Materials and Methods). For method 1 we used the average genome coverage included in the TOPMed metadata file. For method 2, we ‘recomputed’ the average genome coverage based on the number of bases sequenced divided by the standard human genome size (19). Method 3 is the sex-adjusted genome coverage, which is the total number of bases divided by the genome size for females or males. Due to the sex chromosomes, the female genome is 1.02x larger than males. TOPMed also provides an mtDNA-CN metric computed using the program fastMitoCalc (13) and is available for download for TOPMed datasets.

We observed a high correlation between the different mtDNA-CN metrics (all *r* > 0.98, Supplementary Figure S2) and the overall distribution of the mtDNA-CN values were similar for all four CN metrics (Supplementary Figures S3 and S4). Given their similarity, we arbitrarily checked one mtDNA-CN metric, sex-adjusted metric, for any haplotype bias and found no major differences in mtDNA-CN across all haplogroups (Supplementary Figure S3). Although we did note that the overall mtDNA-CN metric is higher in the ARIC cohort compared to MESA, which may be due to a difference in the DNA source.

**Figure 4.**
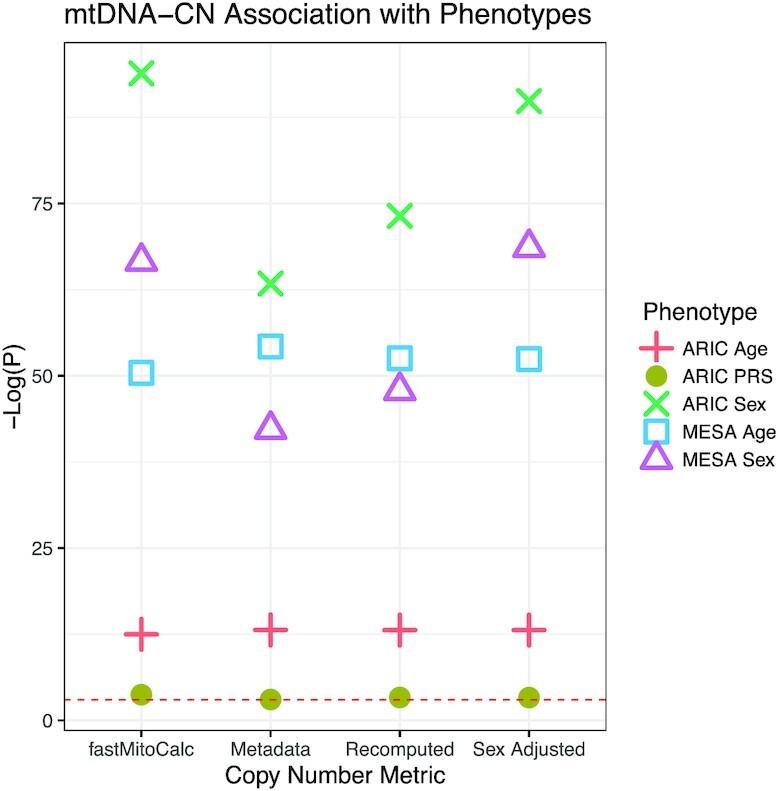
Age and sex association with mtDNA-CN. Linear regression results for mtDNA-CN and age or sex in ARIC (*n* = 3015, left) and MESA (*n* = 4236, right) cohorts. Polygenic risk score (PRS) are only in ARIC. Red dashed line indicates *P*-value of 0.05.

We next sought to understand how strongly each mtDNA-CN metric was associated with known correlated phenotypes (Figure 4). We and others have previously shown that mtDNA-CN measured from peripheral blood decreases with age and is higher in females than in males (39). The mtDNA-CN associations were tested using a liner regression model, adjusted for age or sex, self-reported race, and collection center. As expected, samples from older individuals had a lower mtDNA-CN (*P*-values 4.53 × 10^–06^ sex-adjusted, 4.45 × 10^–06^ recomputed, 5.8 × 10^–06^ metadata, 6.41 × 10^–06^ fastmitocalc) and females had higher mtDNA-CN than males (*P*-value < 2 × 10^–16^ for all metrics). For additional assessment of the mtDNA-CN metrics, we also determined the association of mtDNA-CN with a copy number polygenic risk score (PRS) in the ARIC cohort. PRS was calculated from SNPs identified from GWAS performed in 465 809 individuals in the UK Biobank and Cohorts for Heart and Aging Research in Genomic Epidemiology (CHARGE) (40). When including age, sex, self-reported race and collection center as covariates in our linear regression analysis, all four mtDNA-CN metrics were associated with the mtDNA-CN PRS, with the TOPMed mtDNA-CN provided metric having greatest significance (Figure 4).

To identify the best metric for mtDNA-CN, we ranked each metric based on the strength of the association with known phenotypes as measured by p-values (Supplementary Figure S5). We used the Kendall's *W* test to determine if there was an agreement among the rankings. We observed that there is no significant agreement in ranking, indicating that no mtDNA-CN metric is significantly better than the others. MitoHPC by default will calculate mtDNA-CN as the total number of bases mapped divided by a standard reference genome size (3.03 G), referred to as ‘recomputed’ within this manuscript. In our datasets the difference in effect size between the mtDNA-CN calculations and their association with known phenotypes was subtle, suggesting that the mtDNA-CN metric from WGS is generally robust and the specific calculation should not have a major effect on downstream analyses.

### Characterizing heteroplasmic variants

Mitochondrial SNV heteroplasmies identified by next generation sequencing can be reliably detected at 3% variant allele frequency (VAF) with 1000x chrM coverage (41). We performed our analyses on heteroplasmy SNVs called as low as 3% VAF in our data down-sampled to 2000× coverage. We recommend down-sampling to a higher average coverage if identifying heteroplasmic variants below 3%. However sample quality should be taken into consideration and with higher coverage homoplasmic VAF may cross the heteroplasmic VAF threshold. At 3% VAF, the average heteroplasmic SNV site count was 0.9 and 1.5 for ARIC and MESA, respectively, after excluding outlier counts that were 3 or more standard deviations from the mean. We noticed that samples with heteroplasmic site count greater than 5 have significantly lower mtDNA-CN (*P* = 0.02 in ARIC and *P* < 2 × 10^–16^ in MESA). Even though we did not observe a haplogroup bias with chrM coverage or mtDNA-CN, some haplogroups did have significantly different heteroplasmy counts (Supplementary Figure S6). The L haplogroups have a higher number of homoplasmic variants due to the rCRS reference being most similar to H haplogroups and least similar to L (Supplementary Figure S7).

**Figure 5.**
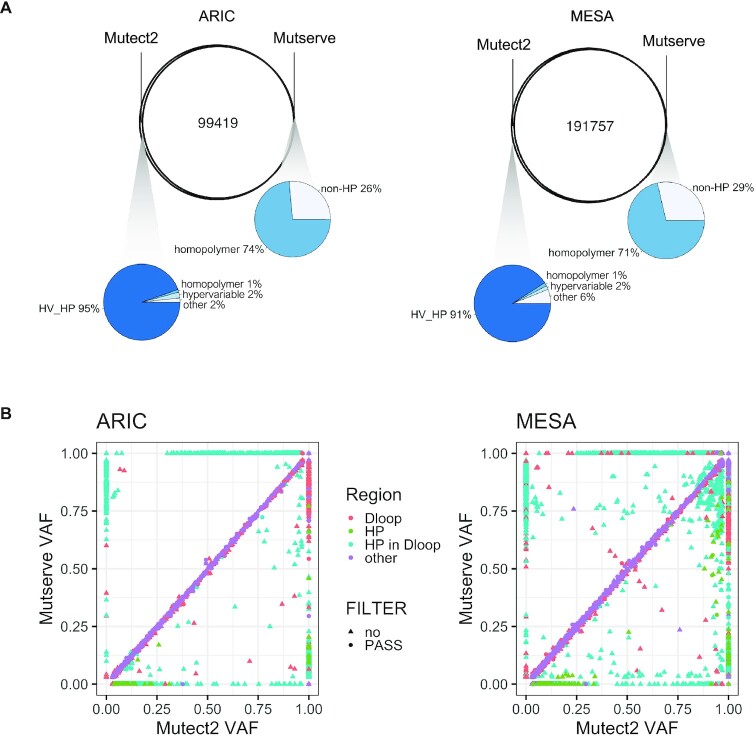
SNVs called by Mutect2 and Mutserve. Venn diagrams show the overlap/differences in the variants called by Mutect2 and Mutserve. The bottom panel shows scatterplots of VAFs of SNVs called at 3% VAF threshold by both software. Colors indicate the location of the variant, in the D-loop (red), in homopolymer (HP) regions (green), in a HP region within the D-loop (blue) or in another region (purple).

We first looked at all SNVs from MitoHPC's first iteration of variant calling. Both Mutect2 and Mutserve had similar distributions of heteroplasmic site counts (Supplementary Figure S8); however, Mutect2 detects more samples as having one or more heteroplasmic sites than Mutserve. For example, Mutect2 identified 2084 samples with one or more heteroplasmic sites in ARIC compared to 1833 by Mutserve. In both ARIC and MESA, the vast majority of variants were identified by both programs, with Mutect2 identifying over 3000 additional variants in ARIC and over 6000 additional variants in MESA (Figure 5A). Of variants that are uniquely identified by either program, only Mutect2 detected variants in chrM hypervariable regions (Figure 5A). When we plotted the VAFs for each SNV in each sample identified by Mutect2 and Mutserve, there were variants where one software called the position a homoplasmy and the other software called the variant a heteroplasmy. This was observed in both cohorts and these variants were almost exclusively in the mitochondrial D-loop (Figure 5B). As a result of the hypervariable regions and poly-C homopolymer tracts in the D-loop, many of the variants identified in this region had low base quality for the alternate allele or had other annotations suggestive of sequencing or technical errors. By performing a comparison of Mutect2 and Mutserve, we found that the genomic substructures of chrM plays a significant role in variant identification. Nevertheless, Mutect2 and Mutserve are largely similar in the variants they identify.

To cross-validate the variants we identified, we determined how many of the variants were also present in the gnomAD v3 database of over 56 000 WGS samples (42). We took 8793 unique chrM homoplasmic and heteroplasmic variants from gnomAD. Of the variants we identified, 93% of sites in ARIC and MESA are also in the gnomAD database. It should be noted that the gnomAD variants had a more stringent frequency cut-off of 10% instead of the 3% used in our study. These results support the conclusion that MitoHPC is likely identifying true variants within the general population, using a lower frequency threshold for defining heteroplasmic variants.

### Detection of true- versus false-positive heteroplasmic variants

Since we identified different variants from the same individual using the different variant calling software, we asked if one software was detecting more false-positive heteroplasmic variants. To test this, we generated 30 simulated datasets representing 30 main haplogroups. Each dataset was simulated to have 150 bp paired-end reads with a random base pair error rate of 0.01. We introduced 43 heteroplasmic sites (8 INDELs and 35 SNVs) into each simulated sample dataset at an average of 18% VAF distribution (range 15–20%). The reads were all mapped using bwa to the rCRS reference. We compared the number of heteroplasmic sites identified by the first iteration Mutect2 (23,24), Mutserve (11) as run by our pipeline, and the online based client Mitoverse (https://mitoverse.readthedocs.io/), which uses Mutserve, and MToolBox (10). MToolBox is the only program that uses the aligner GSNAP (43). We ran the 30 datasets through the different variant callers and counted the number of heteroplasmic sites identified (Table 1). Overall, Mutect2 had the fewest number of false-positives and false-negative calls for both the first and second iteration of variant calling. Mutserve detected more false-positive heteroplasmic sites compared to Mutect2, suggesting that the uniquely identified heteroplasmic variants in Figure 5 were less likely to be real variants, particularly as they occur in homopolymer regions. Since Mutect2 performed the best on our simulated data, we used the Mutect2 variant calls for all analyses moving forward.

**Table 1.** Average number of heteroplasmic sites identified across simulated data—comparison of tools

	SNV + INDEL			SNV			INDEL		
	All sites	False negatives	False positives	All sites	False negatives	False positives	All sites	False negatives	False positives
**100× coverage**									
Mutect2	43.50	1.20	1.70	35.30	0.30	0.60	8.20	0.90	1.10
Mutserve	36.97	9.37	3.33	32.80	3.70	1.50	4.17	5.67	1.83
Mitoverse	38.00	8.70	3.70	34.83	1.77	1.60	3.17	6.93	2.10
MToolBox	50.93	1.10	9.03	41.47	0.17	6.63	9.47	0.93	2.40
**2000× coverage**									
Mutect2	43.03	0.07	0.10	35.03	0.03	0.07	8.00	0.03	0.03
Mutserve	39.17	4.10	0.27	35.10	0.07	0.17	4.07	4.03	0.10
Mitoverse	37.80	6.17	0.97	35.40	0.07	0.47	2.40	6.10	0.50
MToolBox	52.33	0.03	9.37	42.30	0	7.30	10.03	0.03	2.07

### Second iteration Mutect2—calling variants against the sample reference

In its essence, homoplasmy represents inter-individual mitochondrial variation, while heteroplasmy represents intra-individual variation. To leverage this observation, we generate a unique reference for each sample using its own mtDNA consensus sequence generated from homoplasmic sites and major allele heteroplasmic sites called by Mutect2. Heteroplasmic variants are then identified by remapping reads against the sample's unique mtDNA reference sequence. We refer to this as the ‘second iteration’ of heteroplasmy calls. The SNV heteroplasmic site count decreased an average 0.3 counts in ARIC and 0.4 counts in MESA, suggesting a reduction in false-positive heteroplasmies. Notably, some haplogroups had significantly different heteroplasmic site counts (Supplementary Figure S8)

**Figure 6.**
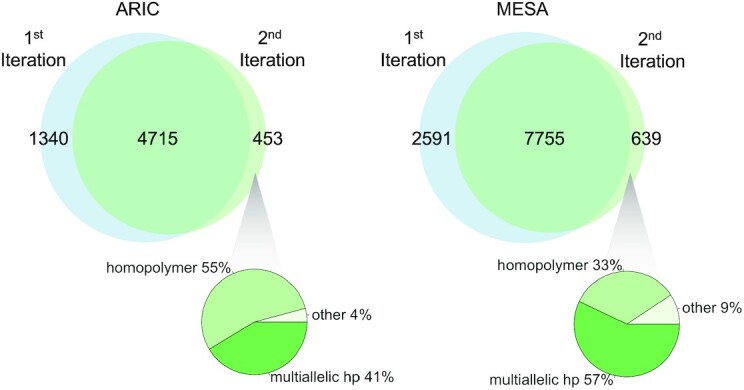
Comparison of heteroplasmic SNV site counts between first and second iteration Mutect2 variant calling (top venn). The pie charts show the percentage of non-allele switch (or ‘other’) variants that were multiallelic, in a homopolymer region, both (MA & HP), or neither (other).

We further investigated the SNVs that differed between the first and second iteration of heteroplasmy. Of these differential SNVs, 96% in ARIC and 90% in MESA are within a homopolymer region (Figure 6). The majority of first iteration unique sites are at positions 302, 310 and 16,183 while the majority of second iteration unique sites are at positions 310 and 16,182. These sites are within the homopolymer regions at positions 296–318 and 16,178–16,193. We also found that 41% and 57% in ARIC and MESA respectively are multiallelic sites. For example, an individual in ARIC had a SNV A > C (3% frequency) at position 302 identified by the second iteration heteroplasmy calling. This sample had two INDELs identified at this same position, AC > A (5%) and AC > ACC (14%). For this sample, in the first iteration, the variants at position 302 were three INDELs, A > AC (6%), A > ACC (74%), A > ACCC (15%). Homopolymer regions complicate variant calling and using MitoHPC we found that these sites have high heteroplasmic variation.

Multiallelic sites do not pass Mutect2 filters but may represent true genetic variation. For example, an individual in MESA has two variants listed at position 3666 allele G > A at 75.9% VAF and G > C at 23.5% VAF for a combined frequency of 99.4%. The G allele must be present at <1%, making this a triallelic site. MitoHPC outputs the full list of variant calls to aid in understanding multiallelic variant sites.

To further validate our approach of calling heteroplasmy after remapping to each samples unique reference mtDNA sequence, we called heteroplasmy on the same 30 simulated datasets described above. At low coverage, running the second iteration of Mutect2 variant calling was the most accurate at identifying the known heteroplasmic sites in our simulated dataset (Table 2). The second iteration identified fewer false positives at 1.7 sites compared to 1.9 sites using just the first iteration of Mutect2. The second iteration of Mutect2 only detected true positive variants at 2000× coverage making it considerably better than other methods at variant calling in our simulated data. We use the second iteration Mutect2 variant calls for further analyses.

**Table 2.** Average number of heteroplasmic sites identified in first versus second iteration variant calling

	All sites	False negatives	False positives
**100× coverage**			
First iteration Mutect2	43.50	1.20	1.70
Second iteration Mutect2	43.10	1.20	1.30
**2000× coverage**			
First iteration Mutect2	43.03	0.07	0.10
Second iteration Mutect2	43.00	0	0

### Heteroplasmy association with age and mtDNA-CN

**Figure 7.**
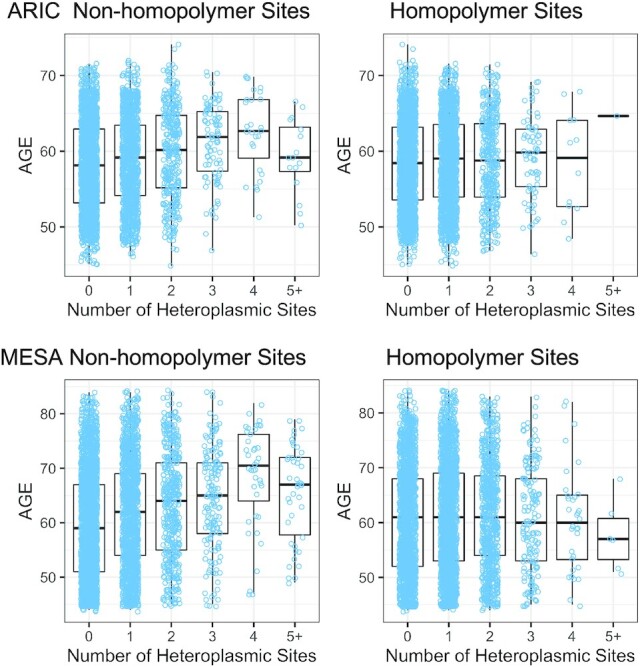
Heteroplasmy by age. Boxplots showing the age distribution of samples categorized by the heteroplasmic site count. X axis corresponds to the count of heteroplasmic sites in a sample. Category ‘’5+’ contains all samples with five or more heteroplasmic sites.

We investigated the association between the number of SNV heteroplasmic sites and age using a negative binomial generalized linear model, adjusted for sex, collection center, and self-reported race. SNV heteroplasmic site counts were from the second iteration of variant calling. Due to the variability of variant calls at chrM homopolymer regions, we counted non-homopolymer and homopolymer heteroplasmic sites separately. As expected, the number of heteroplasmies increased with age and this effect was stronger for the non-homopolymer SNV count (Figure 7, Supplementary Figure S10, and Table 3). The beta estimates were similar for ARIC and MESA, indicating that in both cohorts, we observe a similar age effect on heteroplasmy despite differences in the age distributions of the cohorts (ARIC = 45–74, MESA = 44–84). Additionally, the effect of age on the number of heteroplasmies was 3× larger for non-homopolymer sites but there is still a significant association with homopolymer sites. This suggests that for homopolymer sites there may be a real, biological signal with a reduced effect size due to the inclusion of false-positive calls.

**Table 3.** Association of heteroplasmic site count with age

Age association with heteroplasmy			
Location	Estimate	Std. error	*P* value
ARIC			
Non-homopolymer	3.10 × 10^–2^	5.77 × 10^–3^	8.27 × 10–8
Homopolymer	9.57 × 10^–3^	5.69 × 10^–3^	9.28 × 10^–2^
MESA			
Non-homopolymer	2.91 × 10^–2^	3.20 × 10^–3^	7.53 × 10^–20^
Homopolymer	6.32 × 10^–3^	3.26 × 10^–3^	5.24 × 10^–2^

Age association with the presence of at least one heteroplasmic site. Heteroplasmic sites are counted by location: in a homopolymer region or in a non-homopolymer region for each sample in each cohort.

We investigated the association of SNV heteroplasmic count with sex-adjusted mtDNA-CN. While we observed a general trend that as mtDNA-CN decreases, heteroplasmy increases (Table 4), as previously shown (44), there was extensive heterogeneity of the results. The lack of consistency with respect to effect size in homopolymer and non-homopolymer sites between the two cohorts make it challenging to draw clear conclusions of the results.

**Table 4.** Association of heteroplasmy site count with mtDNA-CN

mtDNA-CN and association with heteroplasmy			
Location	Estimate	Std. error	*P* value
ARIC			
Non-homopolymer	−2.49 × 10^–4^	3.47 × 10^–4^	4.74 × 10^–1^
Homopolymer	−1.34 × 10^–3^	3.46 × 10^–4^	9.98 × 10^–5^
MESA			
Non-homopolymer	−7.89 × 10^–4^	4.73 × 10^–4^	9.49 × 10^–2^
Homopolymer	−2.84 × 10^–4^	4.80 × 10^–4^	5.55 × 10^–1^

mtDNA-CN association with the presence of at least one heteroplasmic site. Heteroplasmic sites were counted by location: in a homopolymer region or in a non-homopolymer region for each sample in each cohort. mtDNA-CN was measured using the sex-adjusted metric.

In addition to homopolymer regions, samples with low mtDNA-CN (<100) may be more likely to yield NUMT variants as false-positive mitochondrial heteroplasmic calls (42). We plotted mtDNA-CN versus heteroplasmy count to visualize the relationship between these two measurements (Supplementary Figure S9). Using a mtDNA-CN cutoff of 100, there were 13/22 samples in ARIC and 3/4 samples in MESA that had at least one heteroplasmy call. Of the three MESA samples with heteroplasmy calls, one sample only had one heteroplasmic site (16092 C > T; VAF 4%) and while that site is not at a known NUMT, it is adjacent to a known NUMT (16093 T > C). Another sample had four heteroplasmic sites, one was a NUMT variant (12612G > A; VAF 3.8%). The third MESA sample had eight heteroplasmic sites all with VAF less than 6%. Six of which were known NUMTs (2706A > G, 7028C > T, 8701A > G, 10398A > G, 11719G > A, 12705C > T). Thus, removing samples with low mtDNA-CN could reduce false-positive heteroplasmies due to NUMT contamination. Setting a higher threshold for heteroplasmic variants can also reduce the number of false-positive heteroplasmies detected.

Previous work by Simone *et al.* (45) identified 585 NUMT regions in human genome build hg18. These regions were lifted over to hg19 and are viewable on the UCSC genome browser. We used a similar approach to identify putative NUMTs in silico in the hg38 genome build. We aligned the rCRS to hg38 and identified 88 putative NUMT regions (Supplementary Table S2). These regions have an average sequence identity of 88.4% to rCRS and an average length of 1012.9 nucleotides. There are 37 putative NUMTs with sequence identity over 90%, of those 25 were <300 nucleotides long. We identified two putative regions that had 100% identity, both were less than 100 nucleotides long. However, most mitochondrial reads do not map to these regions likely due to the fact that the WGS is paired-end 150 and during realignment bwa mem accurately assigns reads overlapping these regions to the nuclear genome.

### INDELs

INDELs were not as consistently identified in the chrM, with high variability between the number of INDELs called by Mutect2 and Mutserve. In MESA, INDEL site count in Mutect2 and Mutserve had an adjusted *r*^2^ of 0.19 for heteroplasmy and 0.06 for homoplasmy. In ARIC, INDEL site count at a 3% VAF threshold had an adjusted *r*^2^ value of 0.17 and 0.05 for heteroplasmic and homoplasmic INDELs, respectively. We further examined the INDELs identified by Mutect2 since Mutserve is not designed for INDEL identification. In ARIC and MESA, respectively, of the >10 000 and >17 000 total INDELs identified occurred at only 119 and 195 unique sites, with 60% in ARIC and 43% in MESA found within 150 bases (the length of an Illumina sequence read) of one of the 9 homopolymer regions on chrM rCRS sequence (Supplementary Figure S11). These regions are prone to PCR, sequencing, and mapping error, and thus, at this time, we are not able to confidently identify INDELs.

## DISCUSSION

Here, we present MitoHPC, a bioinformatics pipeline to analyze mtDNA-CN and heteroplasmy from WGS data. We optimize our pipeline to run quickly and cost-effectively on tens of thousands of samples, a necessity for large scale genomics studies. We built into MitoHPC methods to recover chrM reads at the ends of chrM and at other typically low coverage regions by remapping unmapped reads to a circularized chrM sequence and by remapping reads from NUMTs on chr1 and chr17. These sequences are chrM read ‘sinks’ and with 150 bp paired-end data, we are able to appropriately map them to chrM. We investigated different calculations for mtDNA-CN, and found that there was no significant difference between them. We demonstrate that we detect true population-level SNV variants at 2000× chrM coverage, as shown by our high overlap with variants in the gnomAD database (42). The most novel aspect of MitoHPC is the second iteration variant identification, which calls variants using a sample's unique chrM sequence as the reference. We validate this technique's ability to remove false-positive variants and accurately call true-positives using simulated data. To date, no other heteroplasmy software takes this approach. Our method outperforms existing software in assessing intra-individual heteroplasmic load by identifying heteroplasmic variants against an individual's unique chrM reference sequence rather than a standard reference. We found that homopolymer regions in chrM give most of the variability in heteroplasmy calls and but still have an association with increasing age, although weaker than heteroplasmic sites in non-homopolymer regions. Within our output files, we provide annotated VCFs. Many of these annotations are taken directly from Mutect2 and others are described on our GitHub: https://github.com/ArkingLab/MitoHPC. All-in-all, we demonstrate that our pipeline has increased accuracy and precision in mtDNA variant calling over other existing pipelines and we provide recommendations on how to interpret the data outputs.

We built MitoHPC to run on human data already mapped to hg19 or hg38 reference genomes. However, this pipeline could easily be adopted for other organisms with known mitochondrial variation. There are other interesting facets of mitochondrial genetics that can also be addressed through our pipeline. First, ARIC and MESA used slightly different methods for isolating cells for DNA extraction. It would be interesting to investigate how subtle differences in the blood cell composition could affect the heteroplasmic variants we detect. Second, the analyses presented used the rCRS but we have made RSRS an optional reference genome in our pipeline. This may affect the homoplasmic variants identified, but should have no effect on second iteration of heteroplasmic variant calls.

There are a few limitations to our approach. The work presented here uses paired-end 150 bp sequencing reads. WGS data with shorter read lengths or from different DNA sequencing platforms may perform differently in MitoHPC. Variants identified in homopolymer regions and/or hypervariable regions tend to also have annotations such as strand bias and clustered event. The software identifies these regions as potential sequencing errors. Variants in these regions should be evaluated cautiously or excluded altogether, keeping in mind that many of these variants reside in the D-loop, a region with high sequence variation (46). We leave it to the user's discretion how to handle these variants. We identify heteroplasmic variants as low as 3% VAF using MitoHPC but other software, such as MitoScape (47) may perform better for lower VAF variants and lower mtDNA-CN. INDELs are prone to occur in homopolymer regions. Currently MitoHPC and the heteroplasmy variant identification software are not optimized for INDEL calling. Before assessing the biological significance, it would be advantageous to validate uncertain variants with a secondary method.

It is also important to note that in addition to the Haplocheck output, there are other ways to identify problematic samples in a dataset. Any samples that are outliers for mtDNA-CN or heteroplasmy count should be taken with careful consideration. Samples with a relatively high number of haplogroup specific, hypervariable, or hotspot variants should also be closely inspected. Moreover, samples with a relatively high number of NUMT annotated variants should also be examined prior to downstream analyses. As with any experimental data, it is important to consider results of data analysis within the context of the biology of the samples in question.

In summary, our bioinformatics pipeline MitoHPC captures many features of mitochondrial genetics that are important in understanding the contribution of the mtDNA variation to disease. MitoHPC has high accuracy, high-throughput, and is cost effective, creating a framework for accelerating the analysis of mitochondrial genetics.

## DATA AVAILABILITY

No new sequencing data were created for this study. Sequencing data used in this study is available through dbGaP ((21)): NHLBI TOPMed - NHGRI CCDG: Atherosclerosis Risk in Communities (ARIC) (phs001211.v4.p3); NHLBI TOPMed: MESA and MESA Family AA-CAC (phs001416.v2.p1). All simulated data are available on https://github.com/ArkingLab/MitoHPC.

## Supplementary Material

lqac034_Supplemental_Files

## References

[1] Gorman G.S., Schaefer A.M., Ng Y., Gomez N., Blakely E.L., Alston C.L., Feeney C., Horvath R., Yu-Wai-Man P., Chinnery P.F. et al. Prevalence of nuclear and mitochondrial DNA mutations related to adult mitochondrial disease. Ann. Neurol. 2015; 77:753–759. 25652200 10.1002/ana.24362 PMC4737121

[2] Gorman G.S., Chinnery P.F., DiMauro S., Hirano M., Koga Y., McFarland R., Suomalainen A., Thorburn D.R., Zeviani M., Turnbull D.M. Mitochondrial diseases. Nat. Rev. Dis. Primer. 2016; 2:16080. 10.1038/nrdp.2016.80 27775730

[3] Lake N.J., Compton A.G., Rahman S., Thorburn D.R. Leigh syndrome: one disorder, more than 75 monogenic causes. Ann. Neurol. 2016; 79:190–203. 26506407 10.1002/ana.24551

[4] Goto Y., Nonaka I., Horai S. A mutation in the tRNA(Leu)(UUR) gene associated with the MELAS subgroup of mitochondrial encephalomyopathies. Nature. 1990; 348:651–653. 2102678 10.1038/348651a0

[5] Stewart J.B., Chinnery P.F. Extreme heterogeneity of human mitochondrial DNA from organelles to populations. Nat. Rev. Genet. 2021; 22:106–118. 32989265 10.1038/s41576-020-00284-x

[6] Shoubridge E.A., Wai T. Mitochondrial DNA and the mammalian oocyte. Curr. Top. Dev. Biol. 2007; 77:87–111. 17222701 10.1016/S0070-2153(06)77004-1

[7] Mengel-From J., Thinggaard M., Dalgård C., Kyvik K.O., Christensen K., Christiansen L. Mitochondrial DNA copy number in peripheral blood cells declines with age and is associated with general health among elderly. Hum. Genet. 2014; 133:1149–1159. 24902542 10.1007/s00439-014-1458-9 PMC4127366

[8] Ashar F.N., Moes A., Moore A.Z., Grove M.L., Chaves P.H.M., Coresh J., Newman A.B., Matteini A.M., Bandeen-Roche K., Boerwinkle E. et al. Association of mitochondrial DNA levels with frailty and all-cause mortality. J. Mol. Med. Berl. Ger. 2015; 93:177–186. 10.1007/s00109-014-1233-3 PMC4319988 25471480

[9] Tuppen H.A.L., Blakely E.L., Turnbull D.M., Taylor R.W. Mitochondrial DNA mutations and human disease. Biochim. Biophys. Acta. 2010; 1797:113–128. 19761752 10.1016/j.bbabio.2009.09.005

[10] Calabrese C., Simone D., Diroma M.A., Santorsola M., Guttà C., Gasparre G., Picardi E., Pesole G., Attimonelli M. MToolBox: a highly automated pipeline for heteroplasmy annotation and prioritization analysis of human mitochondrial variants in high-throughput sequencing. Bioinforma. Oxf. Engl. 2014; 30:3115–3117. 10.1093/bioinformatics/btu483 PMC4201154 25028726

[11] Weissensteiner H., Forer L., Fuchsberger C., Schöpf B., Kloss-Brandstätter A., Specht G., Kronenberg F., Schönherr S. mtDNA-Server: next-generation sequencing data analysis of human mitochondrial DNA in the cloud. Nucleic Acids Res. 2016; 44:W64–W69. 27084948 10.1093/nar/gkw247 PMC4987870

[12] Ding J., Sidore C., Butler T.J., Wing M.K., Qian Y., Meirelles O., Busonero F., Tsoi L.C., Maschio A., Angius A. et al. Assessing mitochondrial DNA variation and copy number in lymphocytes of ∼2,000 sardinians using tailored sequencing analysis tools. PLoS Genet. 2015; 11:e1005306. 26172475 10.1371/journal.pgen.1005306 PMC4501845

[13] Qian Y., Butler T.J., Opsahl-Ong K., Giroux N.S., Sidore C., Nagaraja R., Cucca F., Ferrucci L., Abecasis G.R., Schlessinger D. et al. fastMitoCalc: an ultra-fast program to estimate mitochondrial DNA copy number from whole-genome sequences. Bioinforma. Oxf. Engl. 2017; 33:1399–1401. 10.1093/bioinformatics/btw835 PMC5860513 28453676

[14] Puttick C., Kumar K.R., Davis R.L., Pinese M., Thomas D.M., Dinger M.E., Sue C.M., Cowley M.J. mity: a highly sensitive mitochondrial variant analysis pipeline for whole genome sequencing data. 2019; bioRxiv:22 November 2019, preprint: not peer reviewed 10.1101/852210.

[15] Taliun D., Harris D.N., Kessler M.D., Carlson J., Szpiech Z.A., Torres R., Taliun S.A.G., Corvelo A., Gogarten S.M., Kang H.M. et al. Sequencing of 53,831 diverse genomes from the NHLBI TOPMed program. Nature. 2021; 590:290–299. 33568819 10.1038/s41586-021-03205-y PMC7875770

[16] The atherosclerosis risk in communities (ARIC) study: design and objectives. The ARIC investigators. Am. J. Epidemiol. 1989; 129:687–702. 2646917

[17] Bild D.E., Bluemke D.A., Burke G.L., Detrano R., Diez Roux A.V., Folsom A.R., Greenland P., Jacob D.R., Kronmal R., Liu K. et al. Multi-ethnic study of atherosclerosis: objectives and design. Am. J. Epidemiol. 2002; 156:871–881. 12397006 10.1093/aje/kwf113

[18] Li H., Handsaker B., Wysoker A., Fennell T., Ruan J., Homer N., Marth G., Abecasis G., Durbin R. 1000 Genome Project Data Processing Subgroup The sequence alignment/map format and SAMtools. Bioinforma. Oxf. Engl. 2009; 25:2078–2079. 10.1093/bioinformatics/btp352 PMC2723002 19505943

[19] Nurk S., Koren S., Rhie A., Rautiainen M., Bzikadze A.V., Mikheenko A., Vollger M.R., Altemose N., Uralsky L., Gershman A. et al. The complete sequence of a human genome. Science. 2022; 376:44–53. 35357919 10.1126/science.abj6987 PMC9186530

[20] Chen S., Zhou Y., Chen Y., Gu J. fastp: an ultra-fast all-in-one FASTQ preprocessor. Bioinforma. Oxf. Engl. 2018; 34:i884–i890. 10.1093/bioinformatics/bty560 PMC6129281 30423086

[21] Li H., Durbin R. Fast and accurate short read alignment with Burrows–Wheeler transform. Bioinformatics. 2009; 25:1754–1760. 19451168 10.1093/bioinformatics/btp324 PMC2705234

[22] Faust G.G., Hall I.M. SAMBLASTER: fast duplicate marking and structural variant read extraction. Bioinforma. Oxf. Engl. 2014; 30:2503–2505. 10.1093/bioinformatics/btu314 PMC4147885 24812344

[23] McKenna A., Hanna M., Banks E., Sivachenko A., Cibulskis K., Kernytsky A., Garimella K., Altshuler D., Gabriel S., Daly M. et al. The genome analysis toolkit: a mapreduce framework for analyzing next-generation DNA sequencing data. Genome Res. 2010; 20:1297–1303. 20644199 10.1101/gr.107524.110 PMC2928508

[24] Van der Auwera G., O’Connor B 2020; Genomics in the Cloud O’Reilly Media, Inc.

[25] Weissensteiner H., Pacher D., Kloss-Brandstätter A., Forer L., Specht G., Bandelt H.-J., Kronenberg F., Salas A., Schönherr S. HaploGrep 2: mitochondrial haplogroup classification in the era of high-throughput sequencing. Nucleic Acids Res. 2016; 44:W58–W63. 27084951 10.1093/nar/gkw233 PMC4987869

[26] Dür A., Huber N., Parson W. Fine-Tuning phylogenetic alignment and haplogrouping of mtDNA sequences. Int. J. Mol. Sci. 2021; 22:5747. 34072215 10.3390/ijms22115747 PMC8198973

[27] Nussbaum R., McInnes R., Willard H. Thompson & Thompson Genetics in Medicine. 2015; 8th edn. Elsevier.

[28] Lutz-Bonengel S., Niederstätter H., Naue J., Koziel R., Yang F., Sänger T., Huber G., Berger C., Pflugradt R., Strobl C. et al. Evidence for multi-copy Mega-numts in the human genome. Nucleic Acids Res. 2021; 49:1517–1531. 33450006 10.1093/nar/gkaa1271 PMC7897518

[29] Dayama G., Emery S.B., Kidd J.M., Mills R.E. The genomic landscape of polymorphic human nuclear mitochondrial insertions. Nucleic Acids Res. 2014; 42:12640–12649. 25348406 10.1093/nar/gku1038 PMC4227756

[30] Marçais G., Delcher A.L., Phillippy A.M., Coston R., Salzberg S.L., Zimin A. MUMmer4: a fast and versatile genome alignment system. PLoS Comput. Biol. 2018; 14:e1005944. 29373581 10.1371/journal.pcbi.1005944 PMC5802927

[31] Sherry S.T., Ward M.H., Kholodov M., Baker J., Phan L., Smigielski E.M., Sirotkin K. dbSNP: the NCBI database of genetic variation. Nucleic Acids Res. 2001; 29:308–311. 11125122 10.1093/nar/29.1.308 PMC29783

[32] Castellana S., Fusilli C., Mazzoccoli G., Biagini T., Capocefalo D., Carella M., Vescovi A.L., Mazza T. High-confidence assessment of functional impact of human mitochondrial non-synonymous genome variations by APOGEE. PLoS Comput. Biol. 2017; 13:e1005628. 28640805 10.1371/journal.pcbi.1005628 PMC5501658

[33] Danecek P., Bonfield J.K., Liddle J., Marshall J., Ohan V., Pollard M.O., Whitwham A., Keane T., McCarthy S.A., Davies R.M. et al. Twelve years of SAMtools and BCFtools. GigaScience. 2021; 10:giab008. 33590861 10.1093/gigascience/giab008 PMC7931819

[34] Quinlan A.R., Hall I.M. BEDTools: a flexible suite of utilities for comparing genomic features. Bioinformatics. 2010; 26:841–842. 20110278 10.1093/bioinformatics/btq033 PMC2832824

[35] Weissensteiner H., Forer L., Fendt L., Kheirkhah A., Salas A., Kronenberg F., Schoenherr S. Contamination detection in sequencing studies using the mitochondrial phylogeny. Genome Res. 2021; 31:309–316. 33452015 10.1101/gr.256545.119 PMC7849411

[36] Sturk-Andreaggi K., Parson W., Allen M., Marshall C. Impact of the sequencing method on the detection and interpretation of mitochondrial DNA length heteroplasmy. Forensic Sci. Int. Genet. 2020; 44:102205. 31783338 10.1016/j.fsigen.2019.102205

[37] van Oven M., Kayser M. Updated comprehensive phylogenetic tree of global human mitochondrial DNA variation. Hum. Mutat. 2009; 30:E386–E394. 18853457 10.1002/humu.20921

[38] Jeng J.-Y., Yeh T.-S., Lee J.-W., Lin S.-H., Fong T.-H., Hsieh R.-H. Maintenance of mitochondrial DNA copy number and expression are essential for preservation of mitochondrial function and cell growth. J. Cell. Biochem. 2008; 103:347–357. 18072287 10.1002/jcb.21625

[39] Knez J., Winckelmans E., Plusquin M., Thijs L., Cauwenberghs N., Gu Y., Staessen J.A., Nawrot T.S., Kuznetsova T. Correlates of peripheral blood mitochondrial DNA content in a general population. Am. J. Epidemiol. 2016; 183:138–146. 26702630 10.1093/aje/kwv175 PMC4706678

[40] Longchamps R.J., Yang S.Y., Castellani C.A., Shi W., Lane J., Grove M.L., Bartz T.M., Sarnowski C., Liu C., Burrows K. et al. Genome-wide analysis of mitochondrial DNA copy number reveals loci implicated in nucleotide metabolism, platelet activation, and megakaryocyte proliferation. Hum. Genet. 2022; 141:127–146. 34859289 10.1007/s00439-021-02394-w PMC8758627

[41] González M.D.M., Ramos A., Aluja M.P., Santos C. Sensitivity of mitochondrial DNA heteroplasmy detection using next generation sequencing. Mitochondrion. 2020; 50:88–93. 31669622 10.1016/j.mito.2019.10.006

[42] Laricchia K.M., Lake N.J., Watts N.A., Shand M., Haessly A., Gauthier L., Benjamin D., Banks E., Soto J., Garimella K. et al. Mitochondrial DNA variation across 56,434 individuals in gnomAD. Genome Res. 2022; 32:569–582. 35074858 10.1101/gr.276013.121 PMC8896463

[43] Wu T.D., Nacu S. Fast and SNP-tolerant detection of complex variants and splicing in short reads. Bioinforma. Oxf. Engl. 2010; 26:873–881. 10.1093/bioinformatics/btq057 PMC2844994 20147302

[44] Zhang R., Wang Y., Ye K., Picard M., Gu Z. Independent impacts of aging on mitochondrial DNA quantity and quality in humans. BMC Genomics. 2017; 18:890. 29157198 10.1186/s12864-017-4287-0 PMC5697406

[45] Simone D., Calabrese F.M., Lang M., Gasparre G., Attimonelli M. The reference human nuclear mitochondrial sequences compilation validated and implemented on the UCSC genome browser. BMC Genomics. 2011; 12:517. 22013967 10.1186/1471-2164-12-517 PMC3228558

[46] Stoneking M. Hypervariable sites in the mtDNA control region are mutational hotspots. Am. J. Hum. Genet. 2000; 67:1029–1032. 10968778 10.1086/303092 PMC1287875

[47] Singh L.N., Ennis B., Loneragan B., Tsao N.L., Lopez Sanchez M.I.G., Li J., Acheampong P., Tran O., Trounce I.A., Zhu Y. et al. MitoScape: a big-data, machine-learning platform for obtaining mitochondrial DNA from next-generation sequencing data. PLoS Comput. Biol. 2021; 17:e1009594. 34762648 10.1371/journal.pcbi.1009594 PMC8610268

