# Supplementary material for: A bioinformatics pipeline for estimating mitochondrial DNA copy number and heteroplasmy levels from whole genome sequencing data
Source: NAR Genom Bioinform. 2022 May 17;4(2):lqac034. doi: 10.1093/nargab/lqac034 (PMC9112767; doi:10.1093/nargab/lqac034)
Supplement: lqac034_Supplemental_Files [file lqac034_supplemental_files.zip › Supplemental_Materials_submission_REVISED_forSubmission_v2.pdf]

Supplemental Materials

A Bioinformatics Pipeline for Estimating Mitochondria DNA Copy Number and Heteroplasmy Levels from Whole Genome Sequencing Data

Authors: Stephanie L Battle* [1], Daniela Puiu* [2], TOPMed mtDNA Working Group, Joost Verlouw [3], Linda Broer [3], Eric Boerwinkle [4,5], Kent D Taylor [6], Jerome I Rotter [6], Stephan S Rich [7], Megan L Grove [4], Nathan Pankratz [8], Jessica L Fetterman [9], Chunyu Liu [10,11], Dan E Arking[#] [1]

Affiliations:
1 McKusick-Nathans Institute, Department of Genetic Medicine, Johns Hopkins University School of Medicine, Baltimore, MD, USA
2 Department of Biomedical Engineering, Johns Hopkins University, Baltimore, MD, USA
3 Department of Internal Medicine, Erasmus Medical Center, Rotterdam, the Netherlands
4 Human Genetics Center, Department of Epidemiology, Human Genetics, and Environmental Sciences, School of Public Health, The University of Texas Health Science Center at Houston, Houston, TX, USA
5 Human Genome Sequencing Center, Baylor College of Medicine, Houston, TX, USA
6 The Institute for Translational Genomics and Population Sciences, Department of Pediatrics, The Lundquist Institute for Biomedical Innovation at Harbor-UCLA Medical Center, Torrance, CA, USA
7 Center for Public Health Genomics, University of Virginia, Charlottesville, VA, USA
8 Department of Laboratory Medicine and Pathology, University of Minnesota Medical School, Minneapolis, MN, USA
9 Evans Department of Medicine and the Whitaker Cardiovascular Institute, Boston University School of Medicine, Boston, MA, USA
10 Framingham Heart Study, Boston University School of Medicine, Boston, MA, USA.
11 Department of Biostatistics, Boston University School of Public Health, Boston, MA, USA

*co-first authors

[#]Email address of corresponding author: arking@jhmi.edu

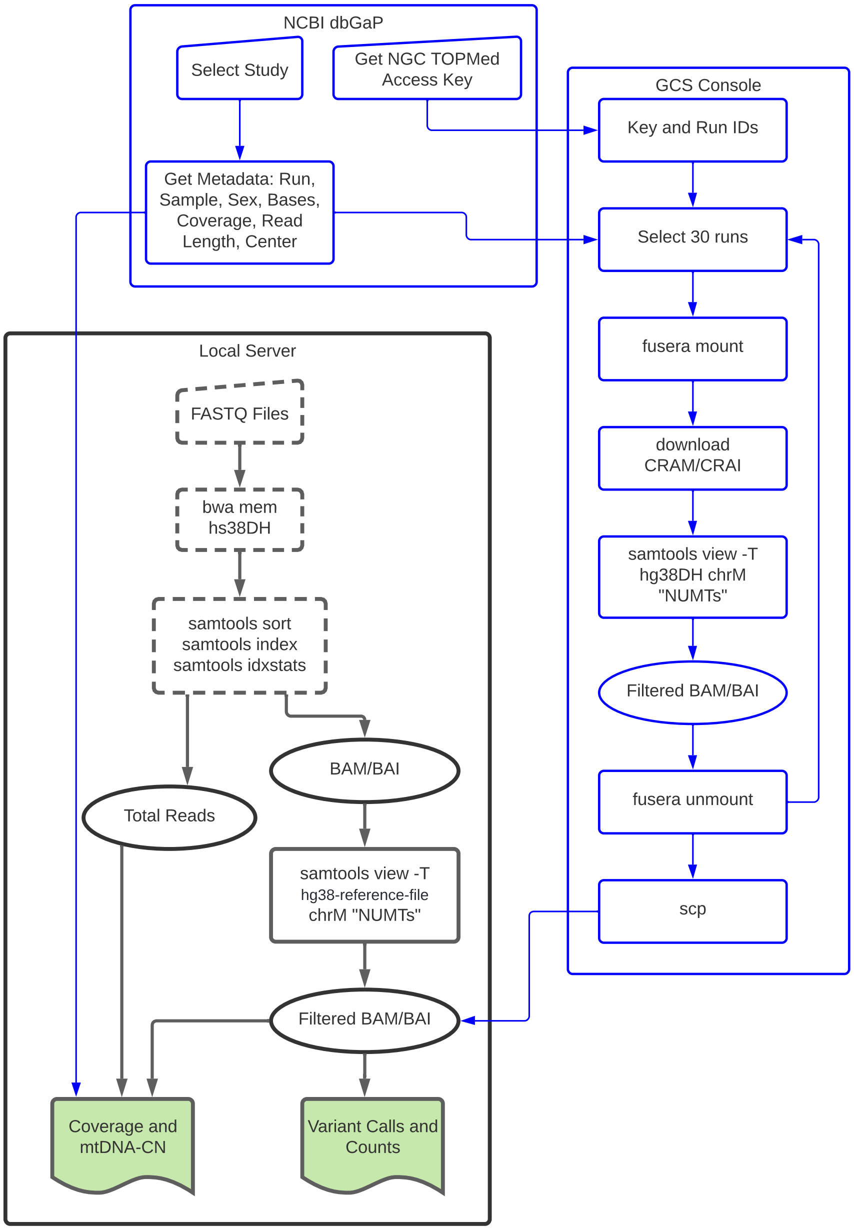

Supplementary Figure 1

Flowchart comparing how accessing TOPMed data differs from samples stored on a local server. Blue boxes indicate the steps specifically used to access TOPMed data. Black boxes show an overview of how to process data stored on a local server. TOPMed WGS data was accessed through NCBI dbGaP. Metadata information downloaded directly to a local server for coverage and copy number calculations. Sample IDs and appropriate access keys were used to process samples on Google Cloud Services (GCS). Samples were processed in batches of 30. We used fusera (https://github.com/mitre/fusera) to access the CRAM and CRAI files, and retrieve reads that map to chrM and the NUMTs on chr1 and chr17 using SAMtools. Files were securely transferred to a local server via scp command. The filtered reads were then run through the pipeline for variant identification.

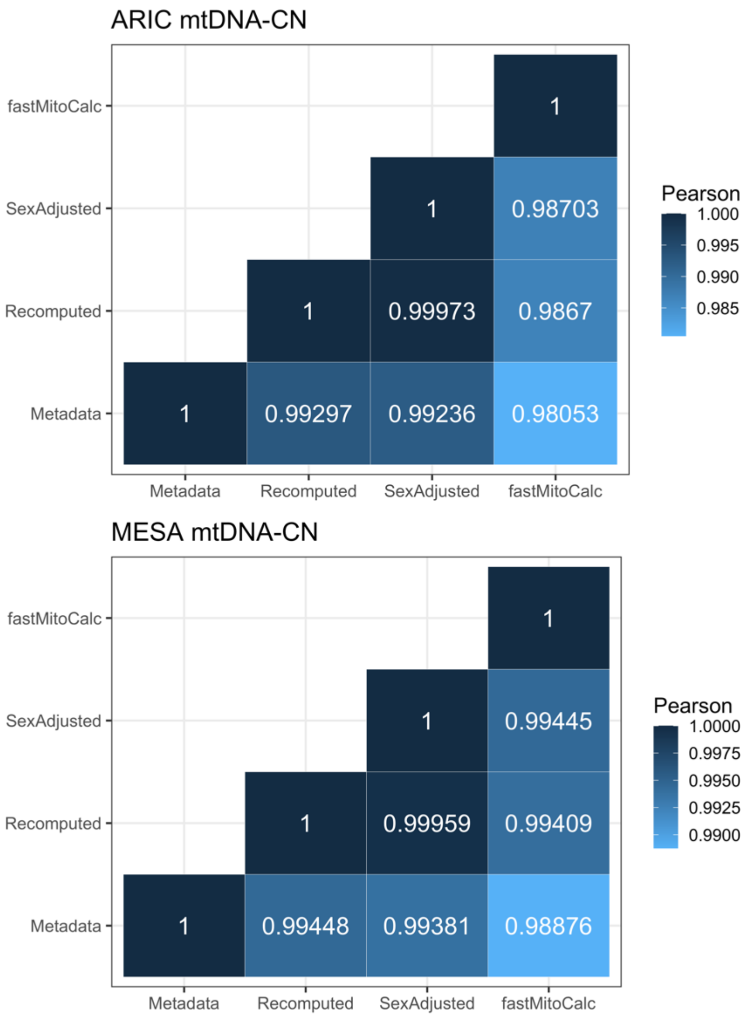

Supplementary Figure 2 Correlation between mtDNA-CN metrics
Pairwise correlation between the different mtDNA-CN calculation metrics is plotted in the matrix for ARIC and MESA cohorts. The Pearson correlation coefficient is written inside the square for each pairwise comparison.

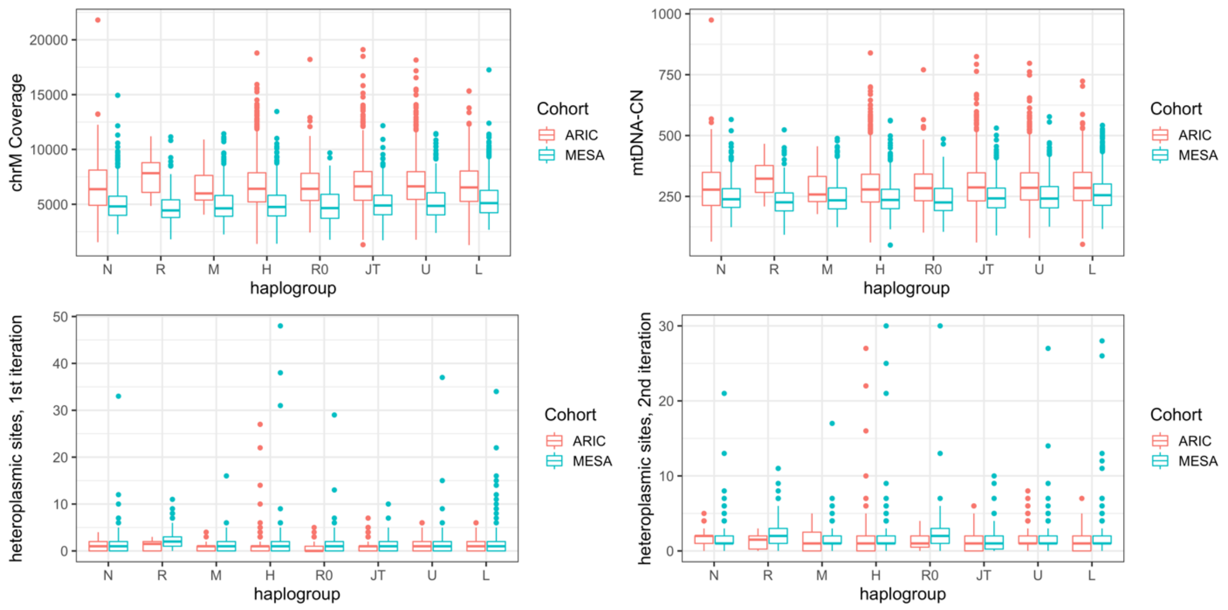

Supplemental Figure 3

Haplogroups are grouped accordingly: H is H only; N contains N, Y, A, S, I, W, X; R contains R1-9, B, P F; M contains M, C, Z, E, D, G, Q; R0 contains R0, HV, V; U contains U, K; JT contains J, T; L contains L0-6. Boxplots for chrM coverage (upper left), mtDNA-CN (upper right), 1st iteration heteroplasmic SNV count (lower left), and 2nd iteration heteroplasmic SNV count (lower right). A linear model, adjusted for age, sex and collection center to test for significant difference among the haplogroups, with haplogroup H being the reference group in the regression model. No significant difference in chrM coverage (upper left plot) was observed between haplogroups in ARIC and only haplogroups R (p-value 0.0004) and L (p-value 4.77e-07) in MESA were significantly different. For mtDNA-CN (upper right), group U (p-value 0.05) in ARIC and groups R (p-value 0.0006) and L (p-value 4.67e-08) in MESA were significantly different. First iteration heteroplasmic SNV count (lower right) is significantly different in group U (p-value 0.0007) in ARIC and group R (p-value 1.96e-08) in MESA. Second iteration heteroplasmic count (lower right) is significantly different in groups N (p-value 0.0009), JT (p-value 0.014) and U (p-value 0.011) in ARIC and groups R (p-value 3.48e-08), R0 (p-value 0.005), and JT (p-value 0.030) in MESA.

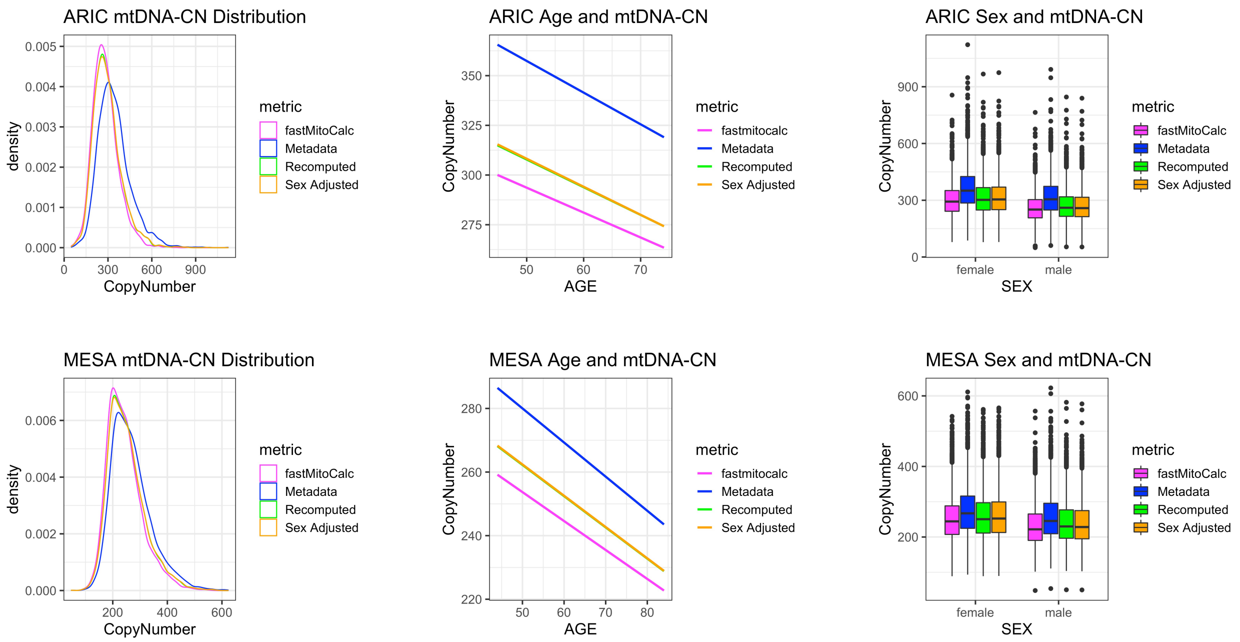

Supplementary Figure 4

Graphs of mtDNA-CN metrics in ARIC (top row) and MESA (bottom row). From left to right: density plot of mtDNA-CN distribution, graph depicting the relationship between sample age (x-axis) and mtDNA-CN (y-axis), and boxplots of mtDNA-CN by sex.

mtDNA-CN Metric	ARIC Age	ARIC Sex	ARIC mtDNA-CN PRS	MESA Age	MESA Sex	Mean Rank	Kendalls W p value
sex adjusted	2	2	3	3	2	2.4	0.89
recomputed	3	3	2	2	3	2.6	
metadata	1	4	4	1	4	2.8	
fastMitoCalc	4	1	1	4	1	2.2	

Supplementary Figure 5 Rankings of mtDNA-CN Strength of Associations. Significance of the ranking by cohort is shown by the Kendall's W p-value.

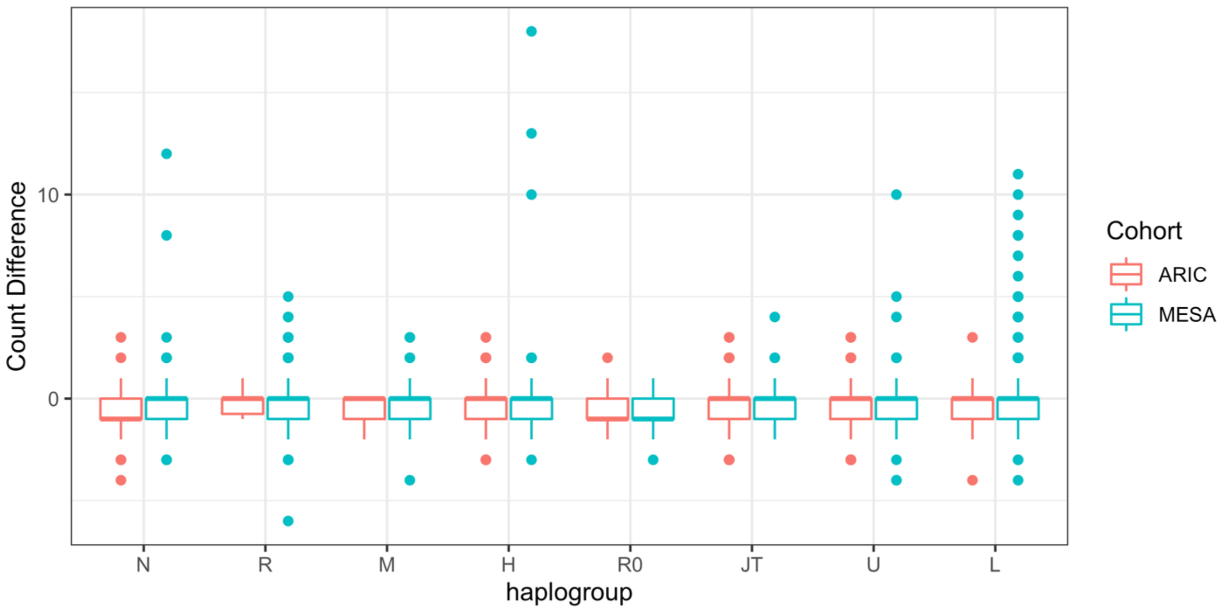

Supplementary Figure 6
Count Difference is defined as the first iteration SNV heteroplasmic site count minus the second iteration heteroplasmic site count. Samples are grouped by haplogroup as defined in Supplementary Figure 2 figure legend.

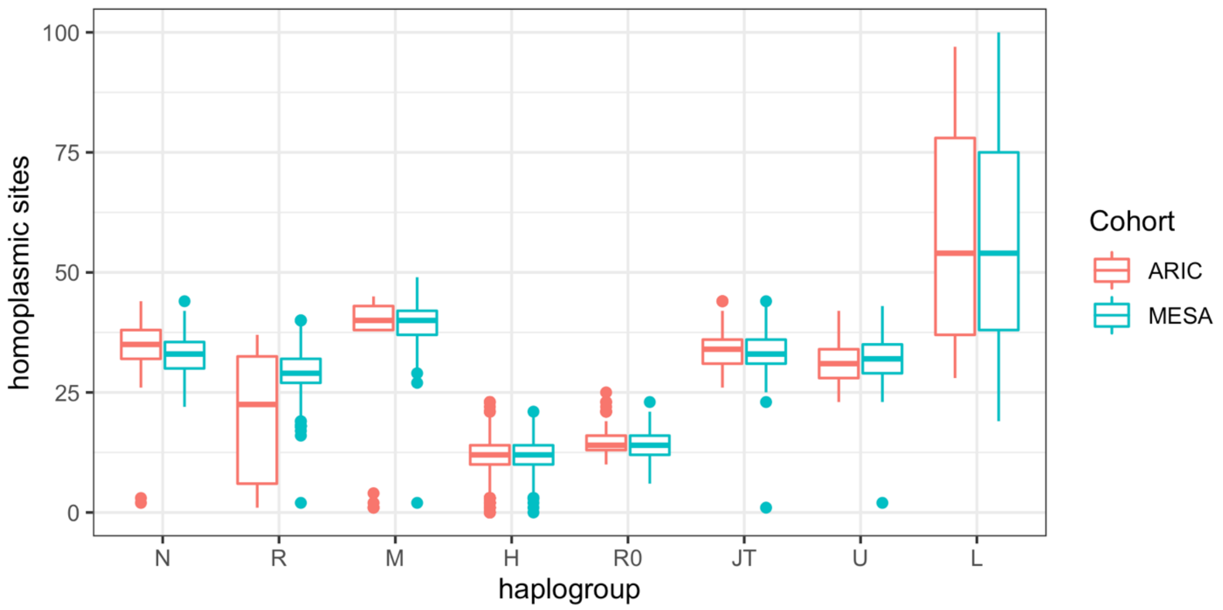

Supplementary Figure 7
Boxplots showing the distribution of chrM sequencing coverage (upper left), mtDNA-CN sex adjusted metric (upper right), heteroplasmic site count from the first iteration variant calling (lower left), and heteroplasmic site count from the second iteration variant calling (lower right). Samples are grouped by haplogroup as defined in Supplementary Figure 2 figure legend.

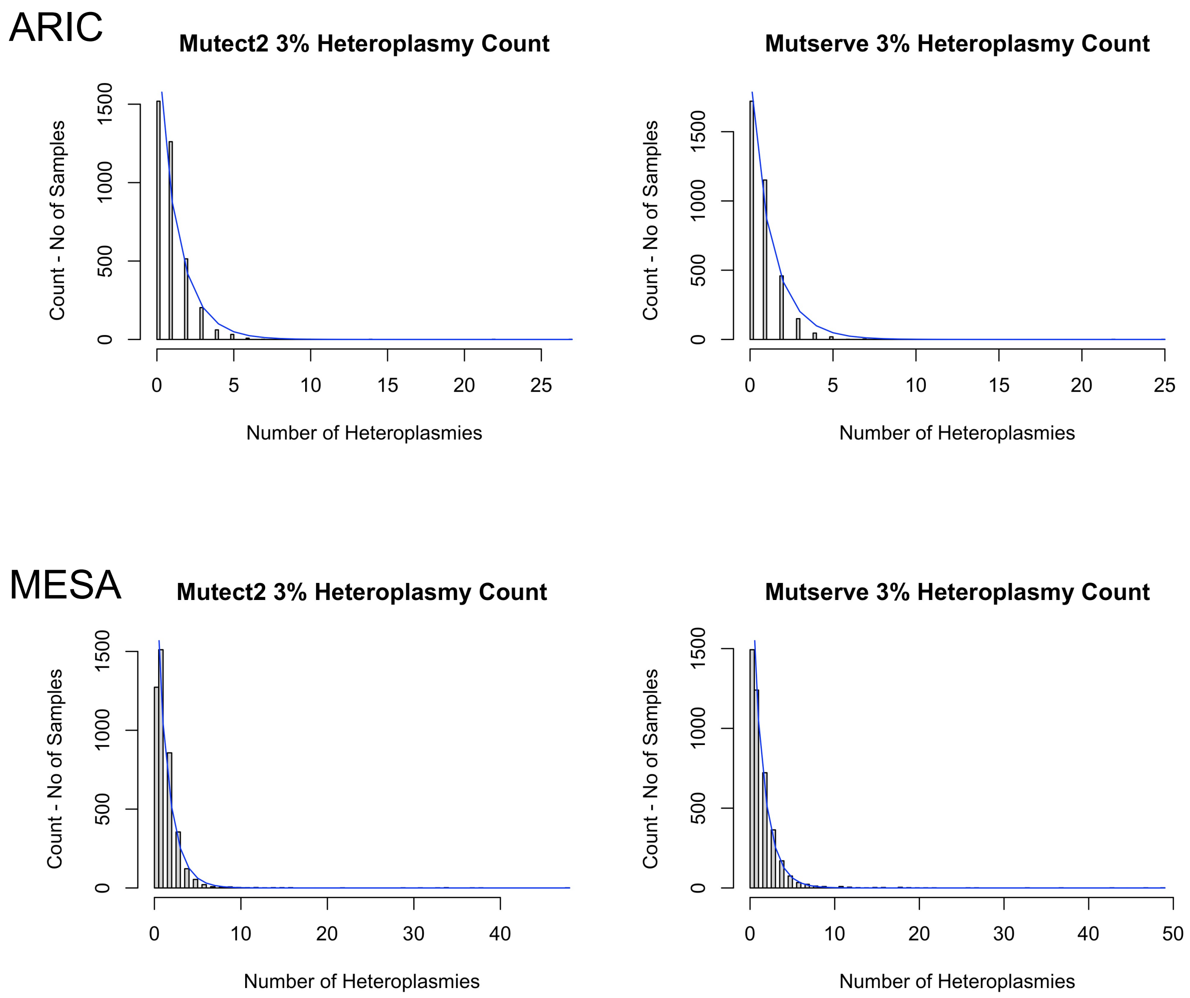

Supplementary Figure 8

Distribution of heteroplasmic SNV site count from Mutect2 and Mutserve identified variants.

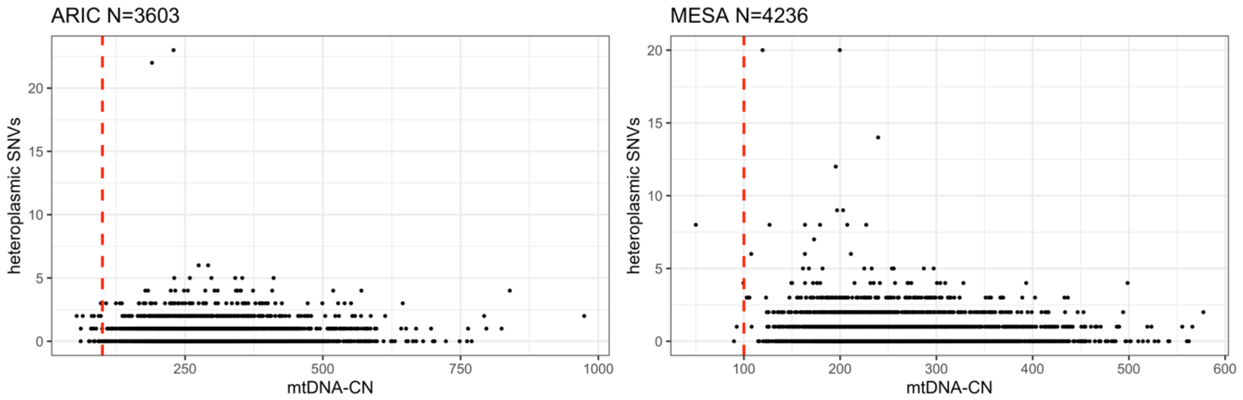

Supplemental Figure 9
Scatterplot of mtDNA-CN and SNV heteroplasmy count for each sample in ARIC or MESA cohorts. Red dashed line is mtDNA-CN = 100.

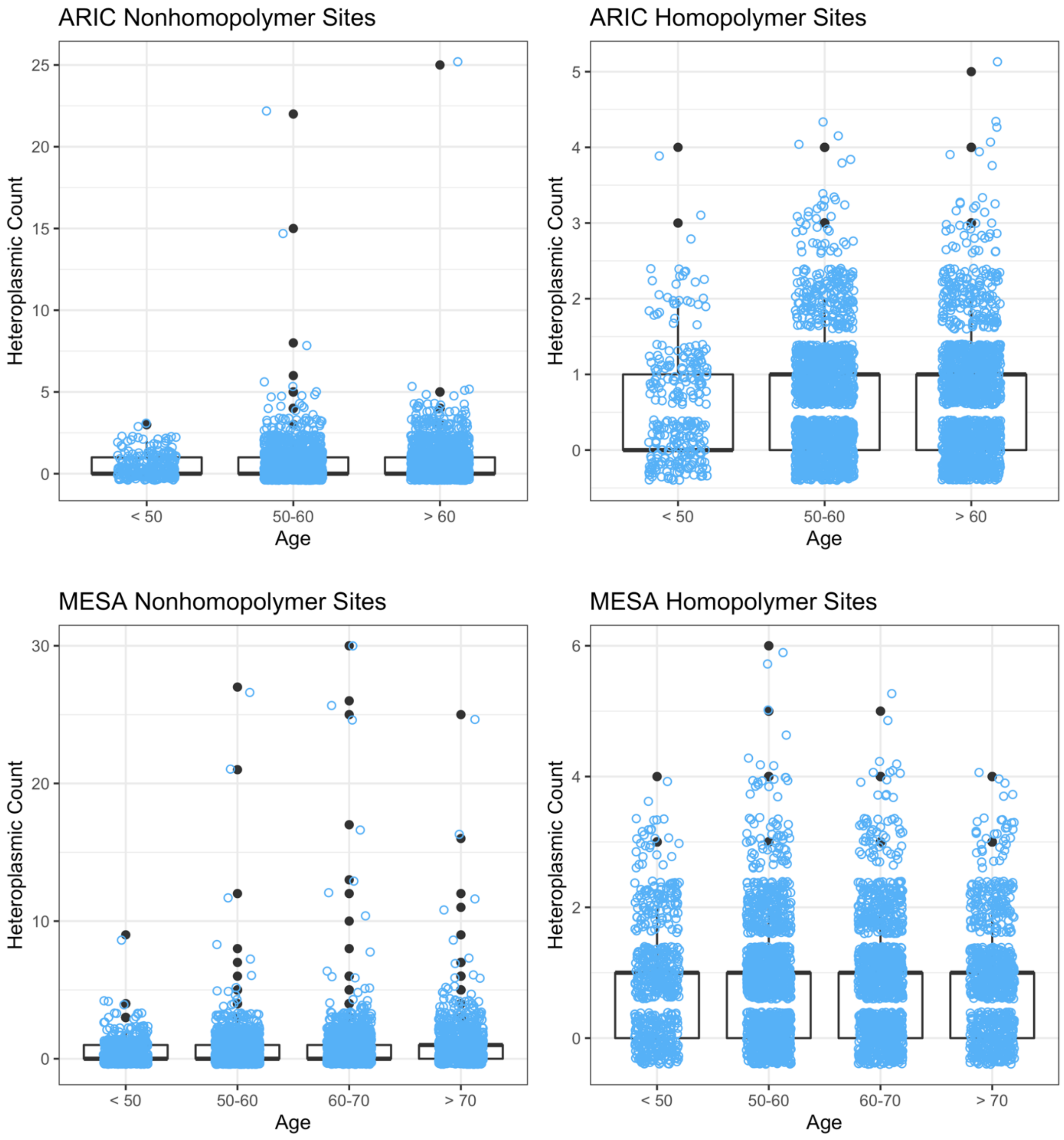

Supplemental Figure 10

Complementary figure to Figure 7 in the main text but with x and y axes switched. Individuals are grouped by age and their SNV heteroplasmy count is plotted along the y axis. A jitter was applied to the points for clarity.

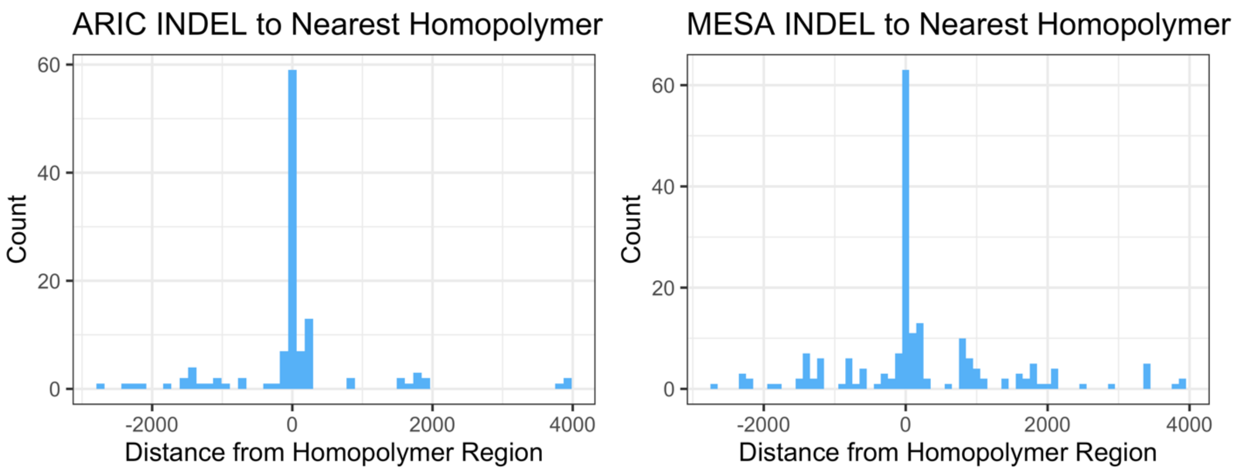

Supplemental Figure 11 Location of INDEL variants relative to homopolymer regions
The x-axis is the distance from an INDEL to its nearest homopolymer region. INDELs that directly overlap a region have a distance of 0.
